# Aesthetic Treatment of the Lips With Hyaluronic Acid Filler: The Multi Vector Lip Technique

**DOI:** 10.1111/jocd.70445

**Published:** 2025-09-11

**Authors:** Marcel Vinícius de Aguiar Menezes, Camila Baioni Garcia Dezanetti, Fernanda Waehneldt Pires Penna, Roberta Simão Lopes Cury, Sabrina Bortoletto Gomes de Aquino

**Affiliations:** ^1^ Private Practice in Plastic Surgery and Dermatology Aracaju Sergipe Brazil; ^2^ Cosmiatry for Doctor Aracaju Sergipe Brazil; ^3^ Private Practice in Dermatology Pato Branco Paraná Brazil; ^4^ Department of Dermatology University of Pato Branco (UNIDEP) Pato Branco Paraná Brazil; ^5^ Private Practice in Dermatology Rio de Janeiro Rio de Janeiro Brazil; ^6^ Department of Dermatology Gamboa Hospital Rio de Janeiro Rio de Janeiro Brazil; ^7^ Private Practice in Dermatology São Paulo São Paulo Brazil; ^8^ Private Practice in Dermatology Lorena São Paulo Brazil; ^9^ Department of Dermatology University of Taubate (UNITAU) Lorena São Paulo Brazil

**Keywords:** filler, hyaluronic acid, lip, vector, VYC‐17.5L

## Abstract

**Background:**

Hyaluronic acid (HA) fillers are a key treatment in the augmentation and rejuvenation of the lips. The novel Multi Vector (MV) Lip technique has been developed using specific injection vectors in geometric forms to shape the lips—allowing precise customization according to the needs of individual patients.

**Aims:**

To describe the MV Lip technique and evaluate its safety and effectiveness in patients treated across five centers in Brazil.

**Methods:**

This was a retrospective analysis of data from adult female subjects. Eligible patients were treated using the HA filler, VYC‐17.5L, in multiple different zones of the lips and perioral area, based on predefined vectors filling geometric shapes.

**Results:**

A total of 253 patients from a range of ethnic backgrounds were included (mean age: 41.0 ± 12.9 years). They received a mean of 1.06 ± 0.21 mL of VYC‐17.5L. On a scale from 1 (poor) to 4 (excellent), mean patient satisfaction with the result was 3.92 ± 0.28. Adverse events were minor cases of edema (*n* = 40; 15.8%) and bruising (*n* = 28; 11.1%). There were no major complications and no cases of vascular occlusion.

**Conclusions:**

The MV Lip technique offers a safe, effective, and reproducible method that is sufficiently versatile to be used across all types of female lips.

## 1 Introduction

The mouth and lips are central to human interaction and play a key role in determining facial attractiveness [1, 2, 3]. In young Caucasian females, the ideal ratio between the vertical heights of the upper and lower lips is around 1:1.6, although these proportions may vary in different ethnicities [4]. Furthermore, this balance typically changes with age. For example, in the upper lip, the cutaneous portion typically lengthens and the volume of the vermilion decreases [4].

Treatment of the lips with hyaluronic acid (HA) fillers is often a key component of facial aesthetic treatment plans. Typically, the focus is on rejuvenation and/or augmentation to try to regain aesthetic ideals as far as possible.

Appropriate product selection is crucial. A filler with intermediate G′ and cohesivity may be preferred, thereby balancing subtle volumizing capacity with the softness and moldability required in a dynamic facial area [5, 6]. A good example is VYC‐17.5L (Juvéderm VOLIFT with Lidocaine, Allergan Aesthetics, an AbbVie Company, Madison, NJ), based on the Vycross technology [7]. VYC‐17.5L has been shown to be safe, effective, and durable in the improvement of the lip and perioral regions [8, 9].

High levels of anatomical knowledge remain essential to the treatment of this area, particularly with regard to the likely locations of key blood vessels [10, 11, 12]. In addition, practitioners must understand the intralabial compartments and their clinical implications. Cadaveric studies have identified 24 distinct lip compartments, whose volumes and sizes each contribute to the overall appearance and shape of the lips [13]. Administration of HA filler in a manner that minimizes the risk of vascular compromise, respects compartments, and considers optimal overall proportions may result in a more harmonious and natural‐looking aesthetic outcome.

Furthermore, practitioners should always maintain a systematic approach to treatment. To achieve this, the lead author developed the Multi Vector Lip technique (MV Lip technique). Many other injectors, some of whom are co‐authors on the present paper, have subsequently adopted the method in their own practice. It is based on the application of an HA filler (VYC‐17.5L) using specific vectors in geometric forms to shape the lips according to desired outcomes—respecting the positions of the labial arteries, fat compartments, and ideal lip measurements. The approach allows for precise customization, thereby meeting the individual aesthetic needs of each patient.

The aim of this paper is to describe the MV Lip technique and evaluate its safety and effectiveness in a group of patients treated across multiple centers.

## 2 Materials and Methods

### 2.1 Study Design and Participants

This was a retrospective analysis of data from 253 patients treated at five different centers in Brazil between 2020 and 2024.

Eligible patients were adult females requiring treatment of the lips using an HA filler. Individuals were excluded if they had: previous treatment with a permanent filler in the lips or perioral area; active autoimmune disease; active infectious disease at the treatment site; any vaccination in the past 30 days; or a psychological condition that could affect treatment. Women who were pregnant or breastfeeding were also ineligible.

The study was conducted in accordance with the Declaration of Helsinki, and all patients provided written informed consent prior to treatment.

### 2.2 Overview of the Technique

The MV Lip technique is based on vectors that fill geometric shapes. These shapes were derived according to the surface anatomy, structures (e.g., tubercles, Cupid's bow, philtral column), and fat‐pad compartments of the lips [13]. Each facilitates assessment for individualized treatment of specific areas in accordance with patient needs.

**FIGURE 1:**
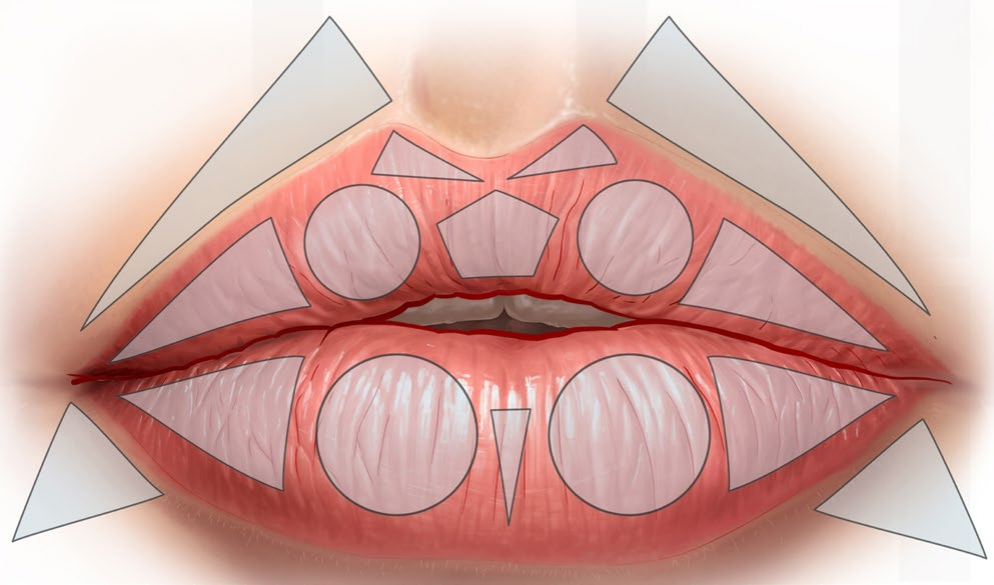
Geometric shapes that define the parameters of the Multi Vector Lip technique.

As shown in Figure 1, the vermilion of the upper lip is divided into two circles (the lateral tubercles), two lateral triangles (for filling the lateral fat‐pad compartments), and one pentagon (central tubercle) underneath two smaller triangles (at the base of Cupid's bow). Similarly, the lower lip vermilion is divided into two circles and two triangles, each symmetrically disposed, and one smaller triangle in the center between the two circles.

Outside the vermilion, the cutaneous part of the upper lip between the nose and the white roll (often the location of “barcode” lines) is divided into two triangles with their base parallel to the philtral column and their apex 0.5–1 cm medial to the nasolabial fold. In addition, below each corner of the mouth, there is a small inverted triangle with its base parallel to the lower lip contouring and its apex at the marionette line.

All treatments in the current analysis were based on VYC‐17.5L, administered using a 30G ½″ needle unless otherwise stated. Suggested product volumes are provided, but these should always be customized to the individual patient, using the minimum amount required to achieve the desired result.

Preferred injection techniques and target depths are described below and in Tables 1 and 2. These were selected with due consideration for the localization of key blood vessels, as observed in anatomical studies [10, 11, 12]. Injection depth is particularly important. An ultrasound study most often mapped the locations of the superior labial artery (SLA) and inferior labial artery (ILA) to the wet mucosal layer, although the SLA was also frequently found in the intramuscular layer and the ILA was sometimes located in the dry mucosa [12]. The mean arterial depths were 5.3 and 4.2 mm in the upper and lower lips, respectively. Hence, augmentation procedures based on superficial injection may help to avoid arterial injury, and this forms the basis of the MV Lip technique. As an additional safety measure, aspiration of ~3 s should be performed every time the needle is inserted, in order to further minimize the risk of vascular placement.

**TABLE 1:** Treatment of the upper lip.

Location	Aim of treatment	Injection device	Point of entering needle or cannula	Injection angle and direction	Deposition of filler	Treatment volume^a^
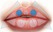	Vermilion eversion; increasing lip height; soft contour	30G needle	Vermilion border	Oblique; lip center to border	Subcutaneous; 3–4 retrograde lines ending with a microbolus	0.02–0.025 mL per line and 0.01 mL in each microbolus
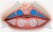	Improving fullness; rejuvenation with soft volumization	30G needle	Vermilion border medial to the corner of the mouth	0° and oblique; medial to lateral	Subcutaneous; 3 single retrograde lines in the circles and in the lateral triangles	0.02–0.025 mL per line (lateral triangle) or 0.05 mL per line (circle)^b^
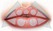	Contouring the upper lip laterally	30G needle	Lateral white roll	Medial to lateral	Superficial subcutaneous; 1 retrograde line	0.025 mL
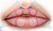	Defining Cupid's bow	30G needle	Vermilion border	Oblique; philtral dimple to G–K point (i.e., medial to lateral along vermilion border)	Superficial subcutaneous; 1 line with retrograde bolus with an optional microbolus at the end	0.025 mL
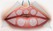	Defining philtral column and G–K point	30G needle	G–K point	Oblique; from up to down	Intradermal; 1 retrograde line	0.025 mL
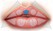	Central upper lip volumization	30G needle	Vermilion border slightly lateral to G–K point	Oblique; lip center to border	Intramuscular; linear retrograde	0.025 mL
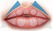	Supporting cutaneous upper lip and softening lip transition	30G needle or 25G cannula	0.5–1 cm above and medial to nasolabial fold	Oblique; medially nasolabial fold to philtrum	Subcutaneous; 3–4 retrograde lines	0.02–0.025 mL per line

^a^ Approximate product volumes are provided, but these should always be customized to the individual patient, using the minimum amount required to achieve the desired result.

^b^ Amount can be tailored according to need for fullness. G–K point, Glogau–Klein point (the highest point of Cupid's bow).

**TABLE 2:** Treatment of the lower lip.

Location	Aim of treatment	Injection device	Point of entering needle or cannula	Injection angle and direction	Deposition of filler	Treatment volume^a^
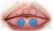	Tubercle definition; vermilion eversion; increasing vermilion show	30G needle	Vermilion border	Oblique; lip center to border	Subcutaneous; 3–4 retrograde lines starting with a microbolus	0.02–0.025 mL per line and 0.01 mL in each microbolus
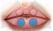	Volumizing inferior tubercle	30G needle	Intramuscular	45° angle; from outside to inside the lip; needle positioned in middle depth of the lip	Intramuscular bolus inside lower lip	Bolus of 0.05–0.1 mL
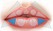	Improving fullness and rejuvenation with soft volumization	30G needle	Vermilion border medial to the corner of the mouth	0° and oblique	Subcutaneous; 3 single retrograde lines	0.02–0.025 mL per line
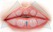	Supporting the medial part of the lower lip	30G needle	Between inferior circles in the midline	Oblique; from up to down	Superficial subcutaneous; 2 retrograde lines forming a V‐shape	0.025 mL per line
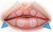	Supporting the corner of the mouth	30G needle or 25G cannula	Medial to the marionette lines	Oblique; medially marionette line to corner of the mouth	Subcutaneous; 2–3 retrograde lines with a microbolus at the mouth corner	0.02–0.025 mL per line and 0.01 mL in each microbolus

^a^ Approximate product volumes are provided, but these should always be customized to the individual patient, using the minimum amount required to achieve the desired result.

**FIGURE 2:**
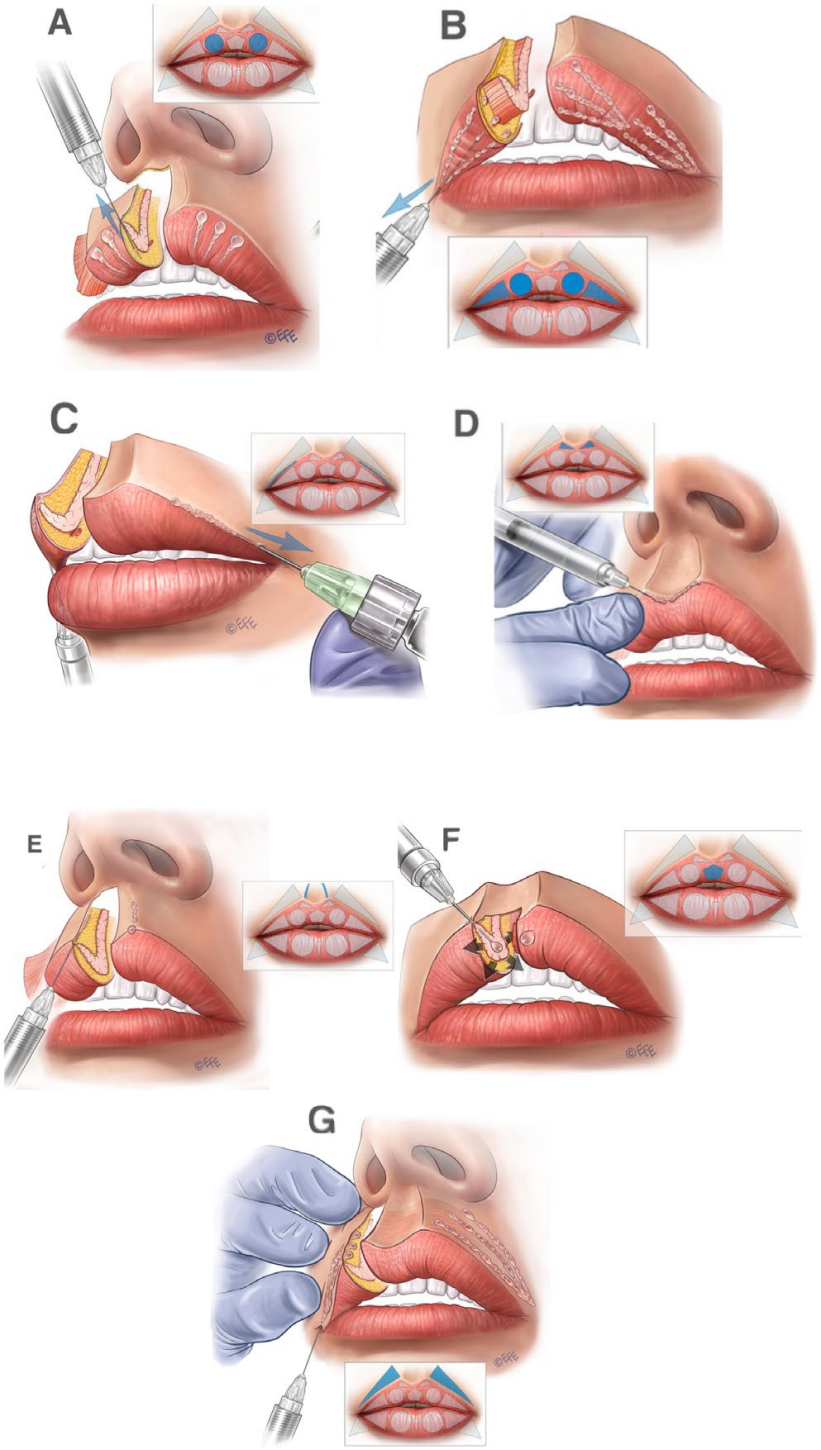
Summary of the treatment sequence in the upper lip. (A–G) Summarize the treatment order as per Figures 5, 6, 7, 8, 9, 10, 11 below. In brief, in (A), vertical vector injections are performed in the subcutaneous plane to volumize the tubercles, create eversion, and define the contour. An outer drop of filler should be left in the lip margin. (B) Lateral vectors based on subcutaneous placement are used to improve fullness and rejuvenate the upper lip. (C) Contouring is done only in the lateral part of white roll with one superficial subcutaneous linear retrograde injection. (D) Linear retrograde injection is made in the superficial subcutaneous plane to refine definition and enhance Cupid's bow. (E) A linear and oblique retrograde injection in the intradermal plane is performed to define the philtral column. A small droplet is placed on the Glogau–Klein point to create natural curvature of the upper lip. (F) An expansion vector is created using an intramuscular bolus to provide volumization without excessive projection. Finally, (G), lateral vectors based on linear retrograde injection are performed in the subcutaneous plane of the cutaneous part of the lips, with the aim of creating a smooth transition between the lip contour and the skin.

**FIGURE 3:**
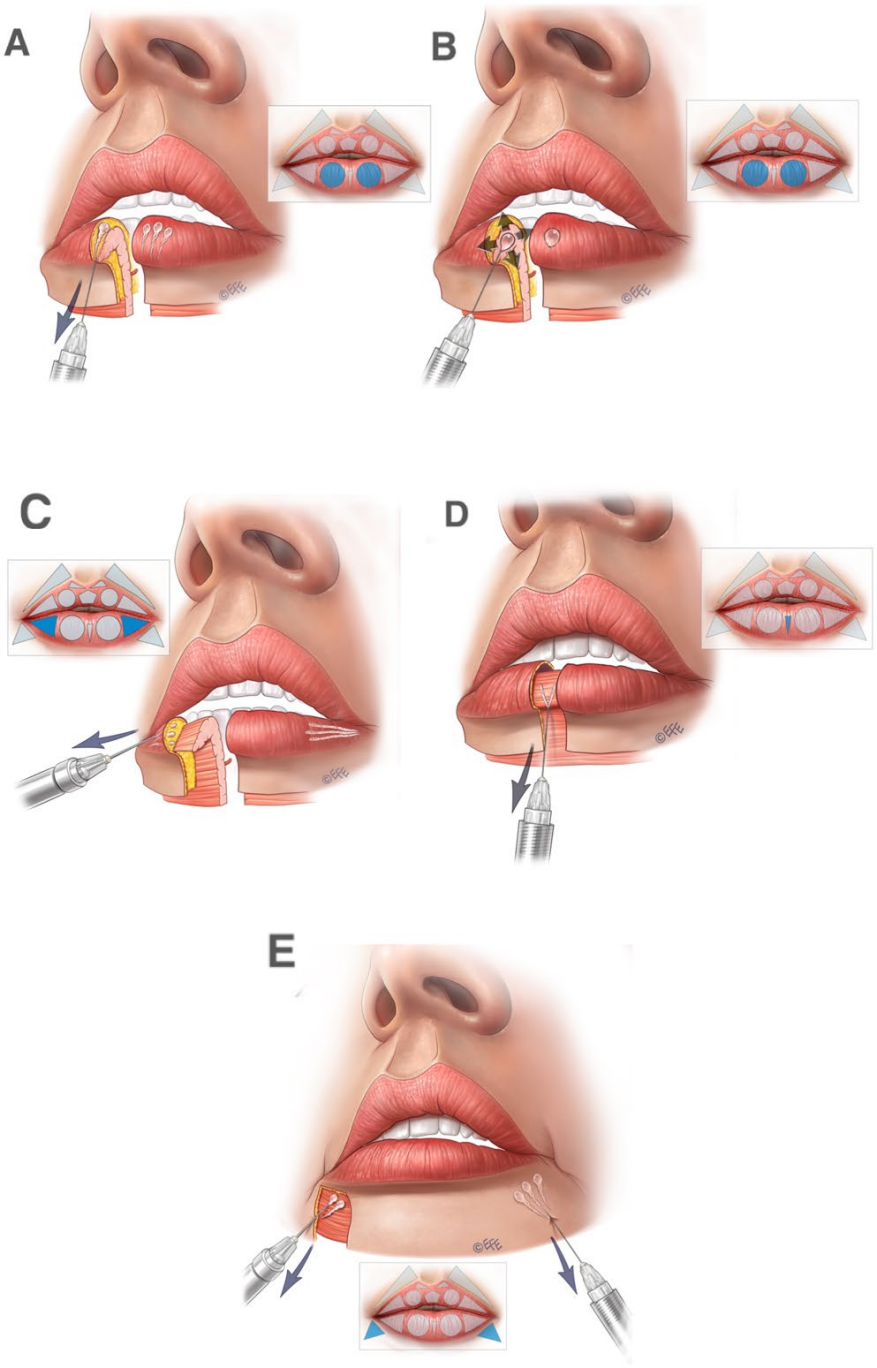
Summary of the treatment sequence in the lower lip. (A–E) Summarize the treatment order as per Figures 12, 13, 14, 15, 16 below. In brief, (A), vertical vectors are injected in the subcutaneous plane. A small droplet of filler should be placed at the transition to the dry mucosa to enhance tubercle definition, promote vermilion eversion, and increase vermilion show. (B), Expansion vectors can be performed using an intramuscular bolus if more volume is needed. (C), Lateral vectors improve fullness and provide rejuvenation of the deflated part of the vermilion. (D), To support the medial portion of the lower lip, one or two retrograde injections may be performed. This approach provides a more natural appearance during smiling, kissing, and speaking. Finally, (E), oblique vectors are injected in the subcutaneous plane for final adjustments, enhancing the harmony between the oral commissures and the overall lip contour.

A full demonstration of the MV Lip technique is provided in Video S1. Separate videos showing individual aspects of the method are cited in the following sections. Summary schematics are also provided for the entire injection sequence in the upper lip (Figure 2) and lower lip (Figure 3).

### 2.3 Upper Lip Treatment

**FIGURE 4:**
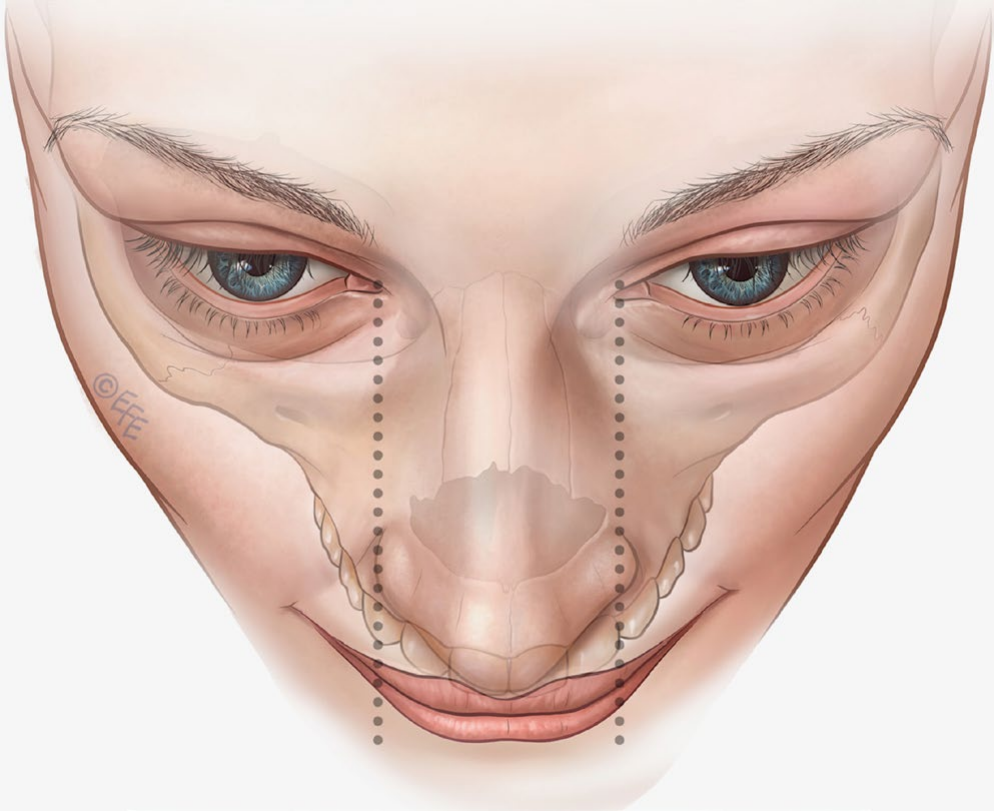
Anatomical landmarks. The tubercles of the upper lip appear more natural when they are positioned close to the center of the lip. The lateral limit of the upper tubercles (upper circles within the MV Lip technique) should be marked between two parallel lines originating at the medial eye canthuses as a fixed anatomical landmark. Another important landmark relates to the incisors. Basing the lateral limit of the upper lip tubercles on the middle of the lateral incisor tooth gives a patient‐specific landmark that allows for individualization to personal anatomy. MV, Multi Vector.

**FIGURE 5:**
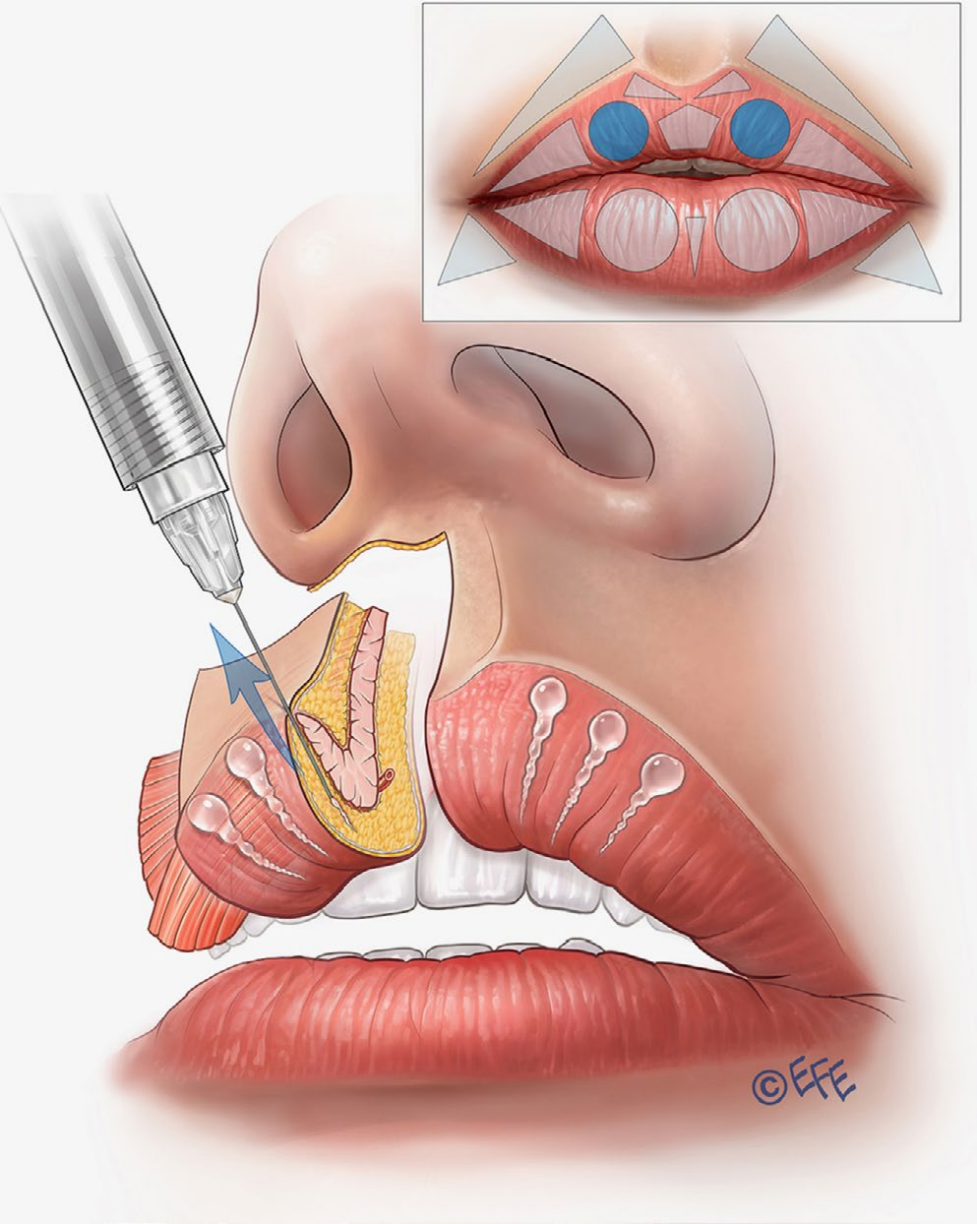
Upper lip: Vermilion eversion, increasing lip height, and soft contouring. Vertical vector injections are performed over the circles that represent the tubercles. It is important to leave a small droplet of filler in the lip margin if there is a need to improve and elevate this area (white roll contour). The lateral limit of the vertical injections should coincide with the middle of the lateral incisor tooth. Vertical vectors are not recommended in the lateral part of the lip because the effect is not sufficiently precise.

The first step is to contour and volumize the lateral tubercles of the upper lip (Table 1). Here, the vector is in the superior circle with 3–4 lines crossing it obliquely in the subcutaneous plane, using linear retrograde injections (around 0.02–0.025 mL per line). The lateral limit is the middle of the lateral incisor tooth (Figure 4), a patient‐specific landmark that therefore allows the method to be individualized to personal anatomy. The injection point is in the vermilion border, with an angled needle, depositing the product from the inferior part of the vermilion in the direction of the cutaneous margin or upper lip border (Figure 5; Video S2). An outer drop of filler may be left on the upper border (0.01 mL per microbolus), but this is not always necessary, for example, when the injector wishes not to elevate the white roll further. Eversion is achieved by exceeding the difference in tissue resistance between skin and mucosa, promoting white‐roll curvature. A key advantage of this approach is that product volumes are small, and the lip does not need to be projected to create eversion, so the results may be more natural. The more vertical the injection angle, the greater the vertical enhancement of the lip.

**FIGURE 6:**
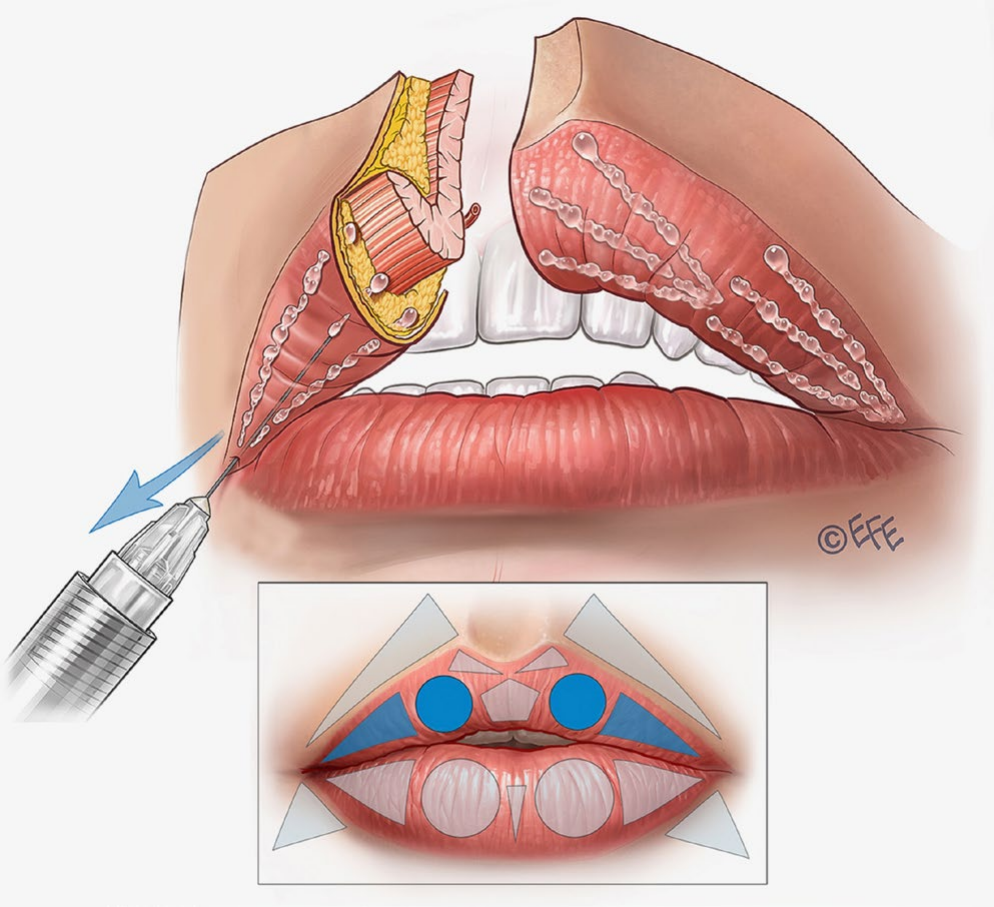
Upper lip: Improving fullness and providing rejuvenation with soft volumization. When injected into the subcutaneous plane of the vermilion, lateral vectors improve fullness and provide rejuvenation of the deflated part of the vermilion. These lateral vectors can be injected over the lateral triangle and also over the circle to further improve the tubercle.

Within the same area, the central fullness of the lips can be improved in some cases with further injections based on three horizontal vectors running retrograde from the medial limit of the tubercle up to the lateral limit (Figure 6; Video S3). Again, the required plane is subcutaneous, using around 0.05 mL of filler per line.

In the next step, if the patient requires volumization, rejuvenation, and/or lateral elongation of the upper lip, a linear vector injection is performed into the superior lateral triangle (Figure 6; Video S3). Some patients require subtle volume in this area, and for best results, the approach can be allied with rejuvenation over the tubercle as per the previous paragraph. The injection point starts at the vermilion border medial to the corner of the mouth, with an angled needle entry, targeting the subcutaneous plane. The product is deposited from the medial to the lateral region by linear retrograde injection, based on 3 lines that converge at the apex of the triangle in the corner of the mouth: one on the upper side, one on the lower side (the longest), and one crossing its central portion. Around 0.02–0.025 mL of filler should be administered per line.

**FIGURE 7:**
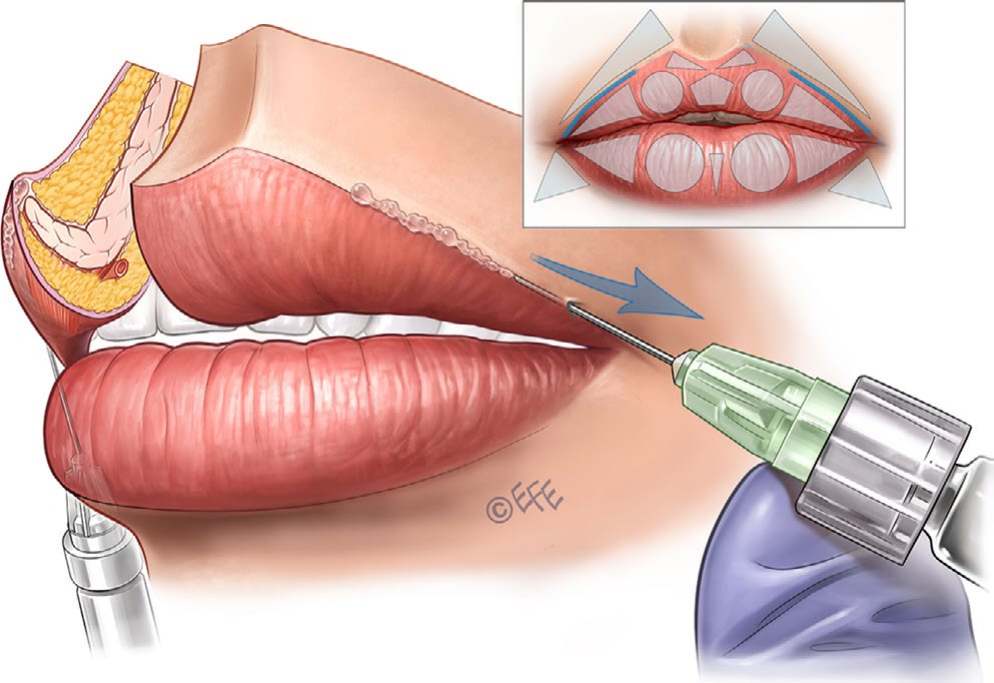
Upper lip: Contouring laterally. In the MV Lip technique, contouring is done only in the lateral white roll because medial contouring is achieved with droplets released with the vertical vectors. MV, Multi Vector.

Lateral contouring of the upper lip—in the anatomical region corresponding to the white roll—is performed by a straight‐line injection into the lateral part of the white roll (above the lateral triangles in the upper lip) (Figure 7; Video S4). Around 0.025 mL of product should be administered on each side.

**FIGURE 8:**
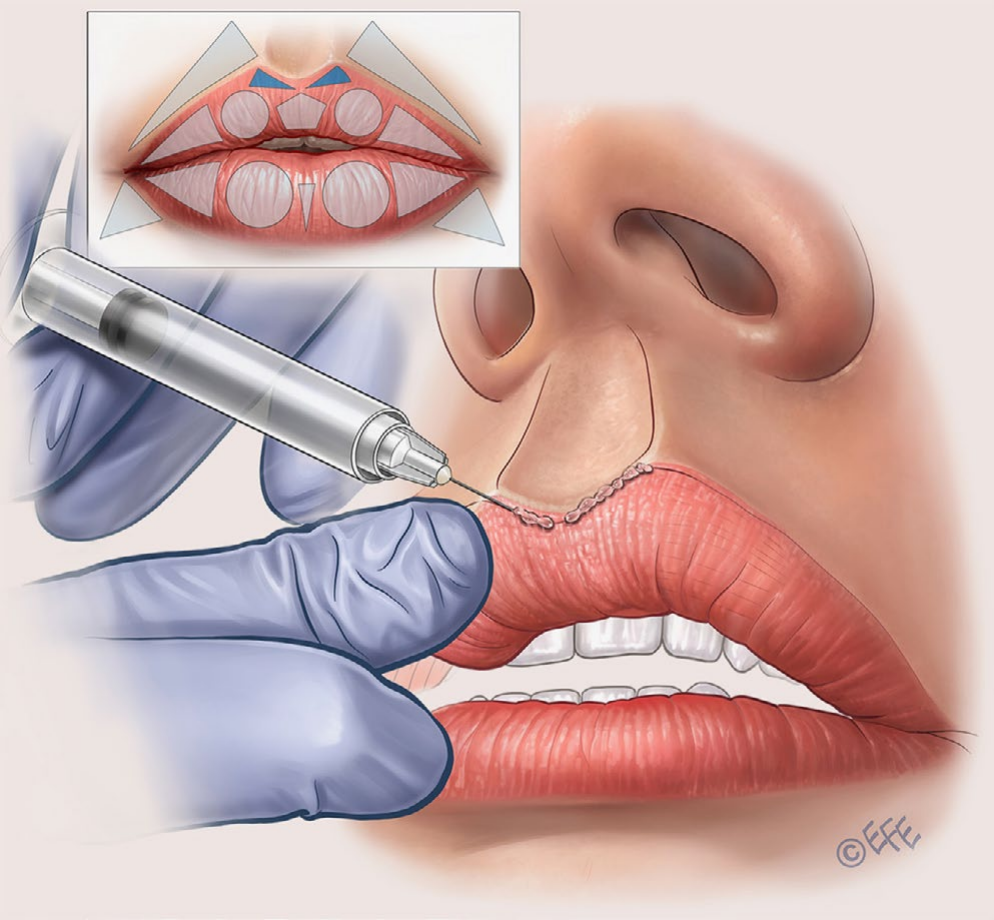
Upper lip: Defining Cupid's bow. Retrograde injection in Cupid's bow creates soft detailing and can be tailored to increase or decrease the width of the central lip using the vectors to close or open the angle.

Cupid's bow is another key area that typically requires filling. To create contour, a vector injection is performed with 1 line of filler administered into the superficial subcutaneous plane using a linear retrograde technique (Figure 8; Video S5). Around 0.025 mL of product is used.

**FIGURE 9:**
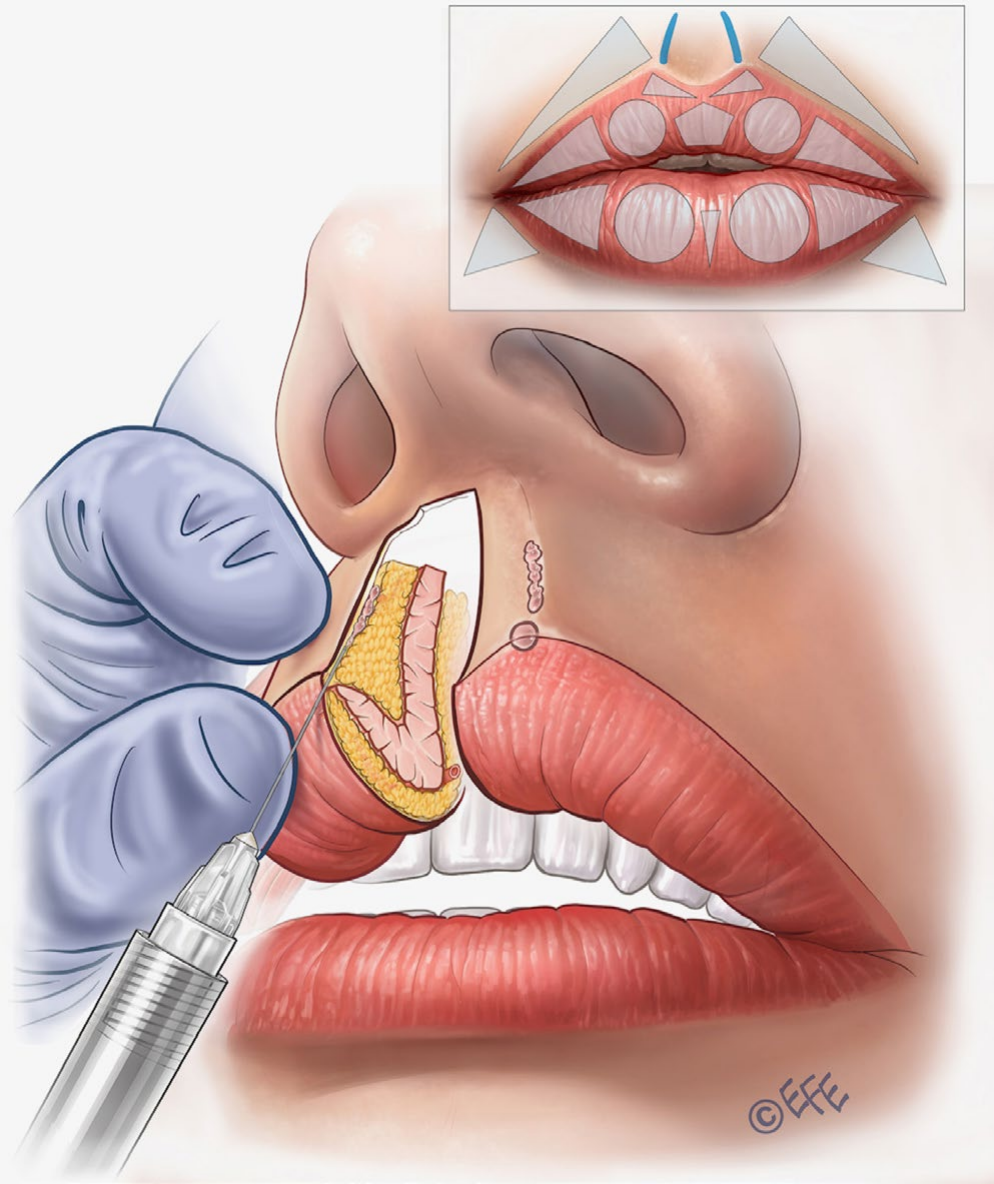
Upper lip: Defining the philtral column and Glogau–Klein point. The philtral columns and G–K points (i.e., the highest points of Cupid's bow) are specially designed within the MV Lip technique. The approach is based on linear retrograde injections that are softly convergent from the mid‐distance between the upper lip and columella. Injections are performed intradermally stopping ~1 mm before the G–K point. A small droplet is then placed on the G–K point. The aim here is to create an interruption that appears more natural. G–K, Glogau–Klein; MV, Multi Vector.

To create a soft, convergent philtral column, the needle is then inserted at the Glogau–Klein (G–K) point (defined as the highest point of Cupid's bow). A linear and oblique retrograde injection is performed in the intradermal plane, starting 2 mm below the nasal columella and ending above the G–K point (Figure 9; Video S6). Typically, 0.025 mL of filler is administered. An important recommendation for added finesse with this vector is that the injector can stop the injection ~1 mm before reaching the G–K point in order to create a small “crease” just above it. Furthermore, at the G–K point itself, a microbolus can be left in order to create some elevation.

**FIGURE 10:**
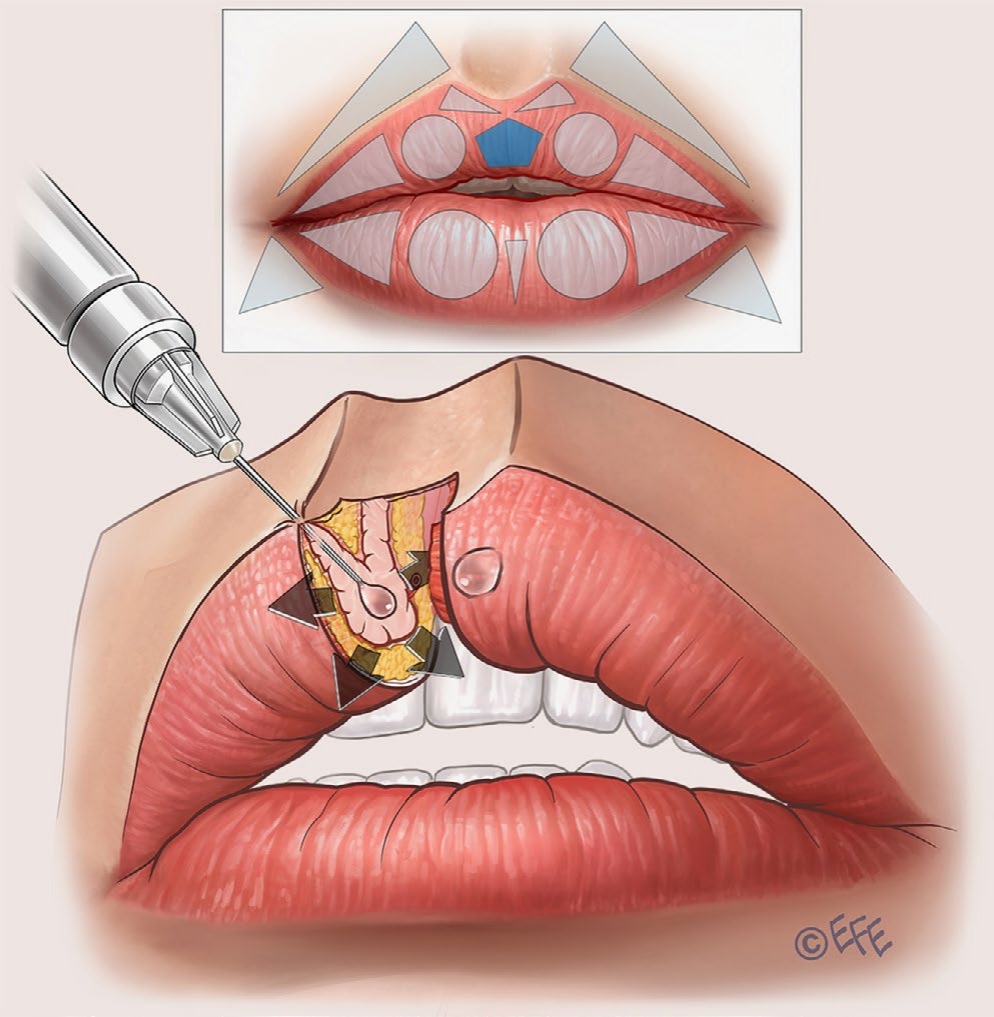
Upper lip: Central volumization. Volumization is achieved based on an expansion vector using an intramuscular bolus. The aim is to give a natural result and provide soft improvement without excessive projection of the medial part of the lip.

For central volumization, the pentagon shape can be treated if necessary. A vector injection is performed with a linear retrograde technique in the intramuscular plane, running from the inferior part of the upper lip to its border, using around 0.025 mL of product (Figure 10). An important practical consideration is that only small amounts of filler should be used in this area—and injectors may want to treat it just to give support to the central part of the lip so that it does not appear “empty,” for example, when smiling or kissing. When injected subtly, this vector can provide volume, while maintaining harmony and naturalness in accordance with lateral treatments.

Overall, if the patient requires treatment in all of these areas, around 0.5–0.6 mL of filler will normally be needed. However, injection volumes should always be individually customized, using the minimum amount of product required to achieve the desired result.

**FIGURE 11:**
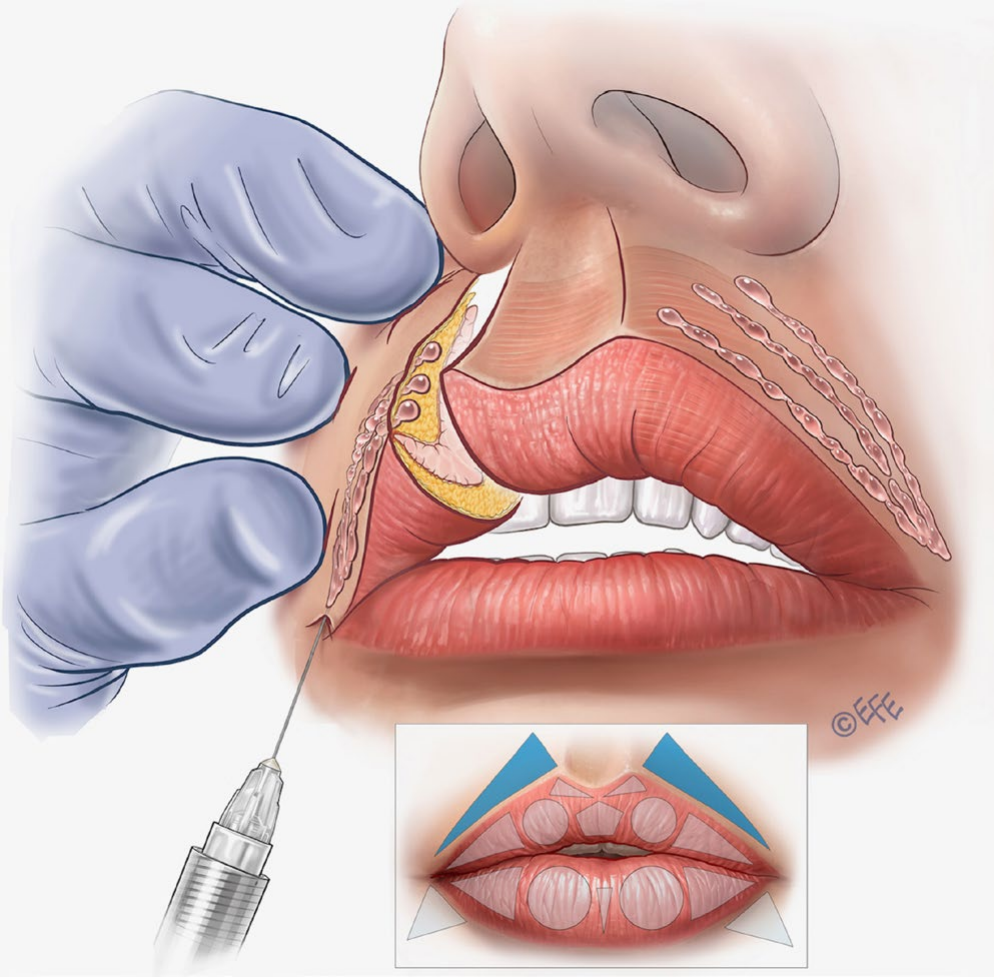
Upper lip: Supporting the cutaneous part and softening the transition. When injected into the subcutaneous plane of the cutaneous part of the lip, lateral vectors increase resistance to the action of the orbicularis oris muscle and can improve “barcode” lines. If performed only close to the lip contour, these injection vectors can create a smooth transition between the lip contour and the skin, which is often desirable in patients who do not want an elevated edge between them.

In some cases, it is also necessary to fill the superior perioral region in the lateral fat‐pad compartments—with the aim of making a soft transition, disguising unwanted perioral lines, and/or replacing fat loss. Injection into the superior lateral triangles (outside the vermilion) is performed via an entry point starting 0.5–1 cm medial to the nasolabial fold, according to the perioral lines of the patient (Figure 11). A 30G needle can be used (Video S7) or alternatively an angled 25G × 38 mm blunt cannula may be preferred (Video S8), reaching the subcutaneous plane laterally to the philtral column. The product is administered by retrograde injection using 3–4 lines that converge on the apex of the triangle. Typically, we use around 0.02–0.025 mL per line, but this should be individualized to the patient to avoid over‐volumization.

When administering linear injections into the subcutaneous plane to fill geometric shapes (such as the superior lateral triangles in the paragraph above), practitioners should consider massage of the treatment area to optimize distribution of the filler. This can be particularly valuable in optimizing the naturalness of results.

### 2.4 Lower Lip Treatment

**FIGURE 12:**
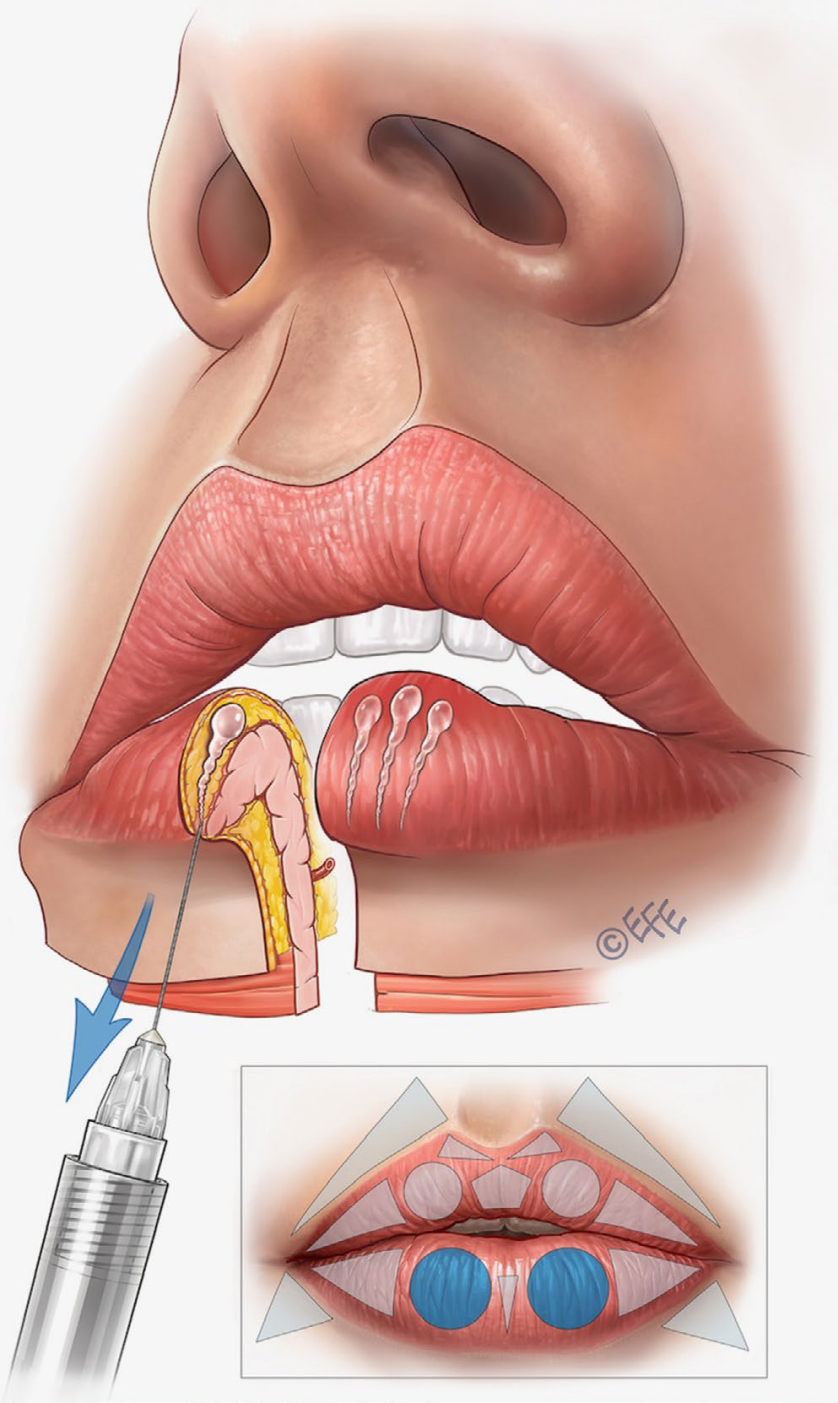
Lower lip: Tubercle definition, vermilion eversion, and increasing vermilion show. Vertical vectors are injected over the circles that represent the inferior tubercles. It is important to leave a small droplet of filler within the subcutaneous plane at the transition of the wet and dry mucosa. A special tip is to make 3 (or even 4) vertical vectors, leaving the central vector a little higher than the lateral ones. This will create a circular design to give a more natural look to the inferior tubercle. If more volume is needed, these injections can be followed by an intramuscular bolus (expansion vector), as shown in Figure 13.

**FIGURE 13:**
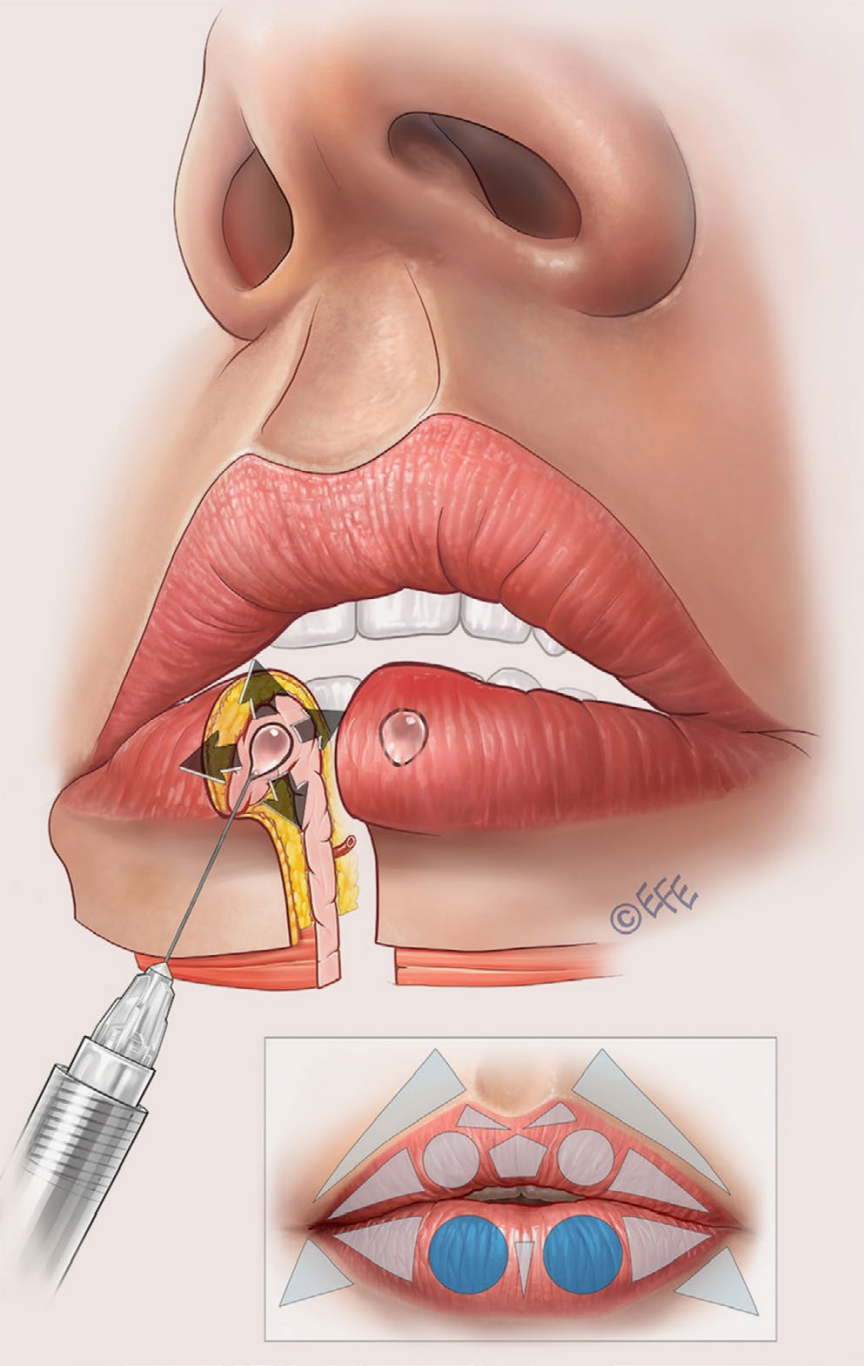
Lower lip: Volumizing the inferior tubercle. If more volume is needed in the inferior tubercles, expansion vectors can be performed using an intramuscular bolus in each.

The starting point here is the two circles that represent the inferior lip tubercles (Table 2). Vertical vector injections are performed based on 3–4 lines inside each circle, using around 0.02–0.025 mL of product in each line and a retrograde technique (Figure 12; Video S9). The target layer is the subcutaneous plane. To enhance the tubercle, a microbolus of ~0.01 mL should be left at the beginning of the retrograde injection in the transition of the wet and dry mucosa. The middle line should be higher than the others to promote tubercle curvature. According to the assessment of the individual patient, supplementation with an intramuscular bolus of ~0.05 to 0.1 mL of product can be performed in the middle of the tubercle, with an injection angle of 45° (Figure 13; Video S10).

**FIGURE 14:**
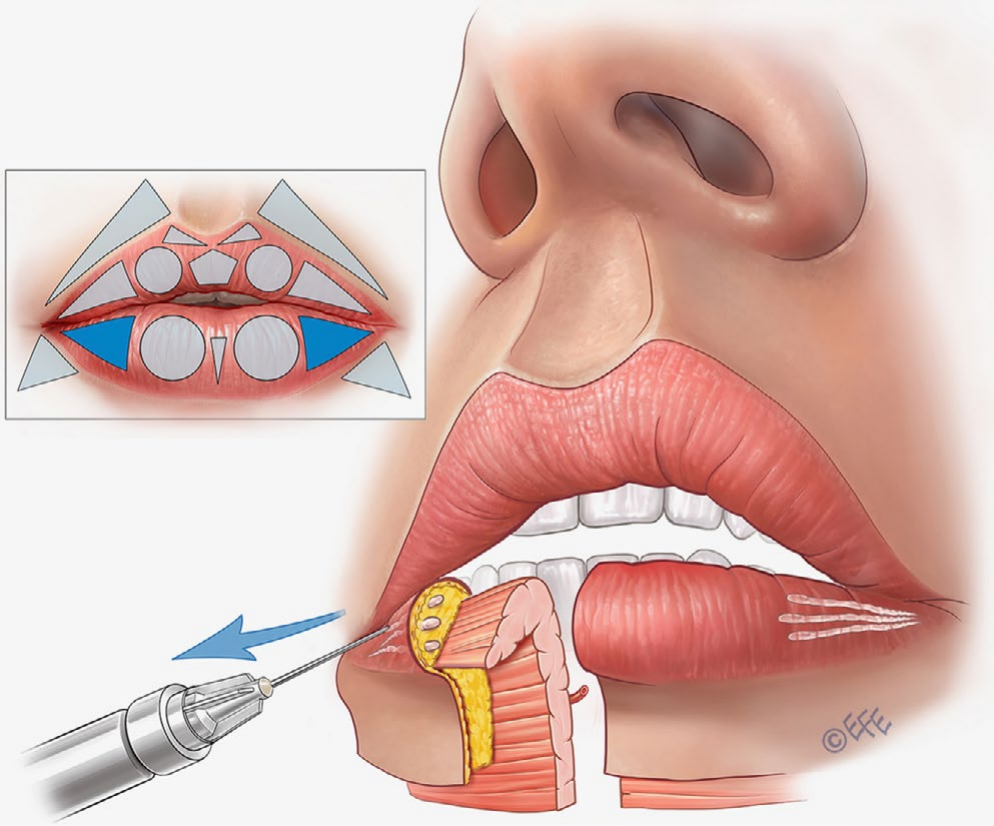
Lower lip: Improving fullness and rejuvenation with soft volumization. When injected into the subcutaneous plane of the vermilion, lateral vectors improve fullness and provide rejuvenation of the deflated part of the vermilion. It is important to appreciate that the more the lateral vectors are injected, the more attention is called to the width of the lip. If the patient has large lips, these vectors may be less important compared with the central ones.

To fill the lateral triangle, horizontal vector injections are performed in a similar way to the upper lip (Figure 14). A total of 3 lines is typically administered in the subcutaneous plane, using around 0.02–0.025 mL of product per line and a retrograde technique from medial to lateral. These lines converge on the apex of the triangle—one on the upper side, one on the lower side, and a third crossing the central portion of the lip.

**FIGURE 15:**
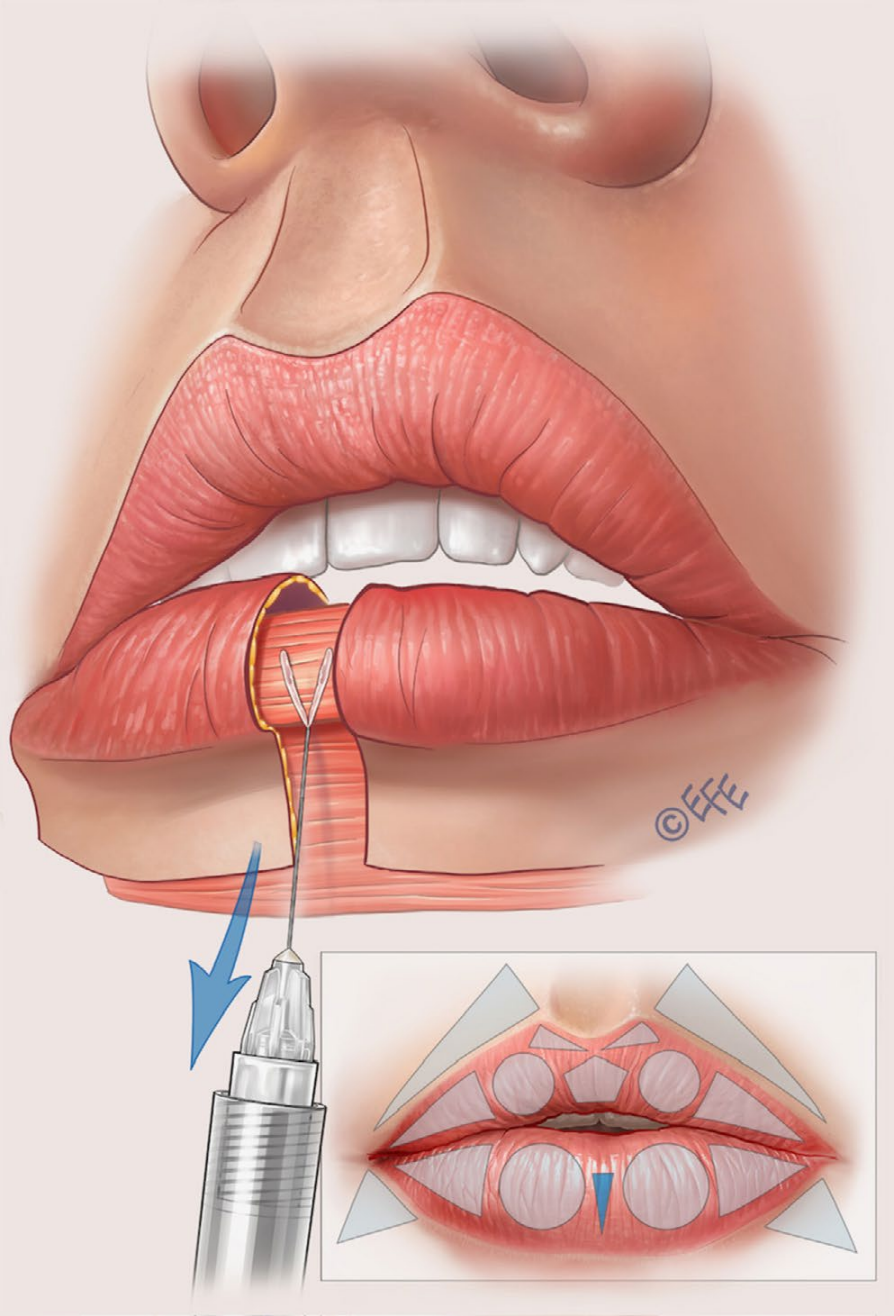
Lower lip: Supporting the medial part. One or two retrograde injections can be made to provide some resistance in this area. Treatment gives a more natural aspect when the patient smiles, kisses, or speaks, to help avoid unnatural expressions.

When injecting the inferior tubercles, the center of the lower lip may sometimes become less volumized. Two linear retrograde injections (0.025 mL per line) can be performed into the subcutaneous layer forming a V‐shape—with the apex in the vermilion border of the lower lip and finishing before the transition between the wet and dry mucosa (Figure 15; Video S11). This is important to fill the central triangle and soften the difference with more lateral areas.

Overall, a similar amount of product is typically used in the lower lip compared with the upper lip.

**FIGURE 16:**
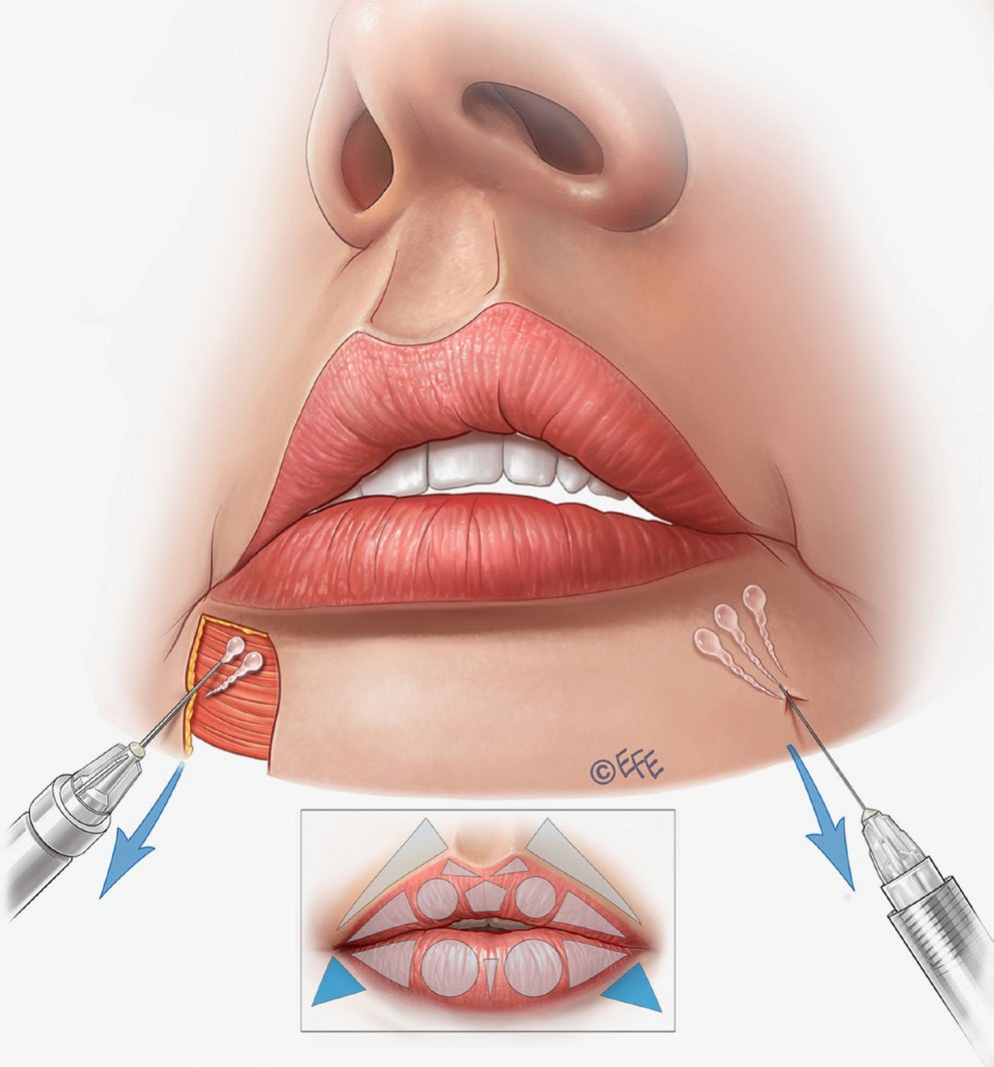
Lower lip: Supporting the corner of the mouth. These vectors are injected to give support outside of the lip with fanning injections in a triangle that shows fat loss in some cases. The aim is to create a natural transition between the lip and the surrounding areas.

Finally, to angle the corner of the mouth upwards, and adjust a “sad” aspect to the lips or correct marionette lines, the triangles under the mouth corner (outside the vermilion) can be treated. Two to three retrograde injections are performed in these triangles, leaving a microbolus near the corner of the mouth (Figure 16). Typically, 0.02–0.025 mL of product is used per side, but this should always be customized depending on needs. A 25G cannula or 30G needle may be used.

### 2.5 Assessments

For all patients in the current analysis, demographic data were collected at baseline and adverse events (AEs) were recorded throughout 9–12 months of follow‐up. Patient and physician satisfaction with the results were assessed 1–3 months posttreatment using a four‐point scale (1, poor; 2, moderate; 3, good; 4, excellent).

### 2.6 Statistical Analysis

Descriptive statistics are provided throughout, including mean and standard deviation for continuous variables, and frequency and percentage for categorical variables.

## 3 Results

A total of 253 female patients were included in the present analysis. The mean age was 41.0 ± 12.9 years, and they were highly ethnically diverse (Table 3).

**TABLE 3:** Patient and treatment characteristics.

Characteristic	Patients (*N* = 253)
Sex, female, *n* (%)	253 (100)
Age, years, mean (SD)	41.0 (12.9)
Ethnicity, *n* (%)	
Hispanic/Latin	134 (53.0)
Caucasian	77 (30.4)
Black	32 (12.6)
Asian	6 (2.4)
Indigenous	4 (1.6)
VYC‐17.5L treatment volume, mL, mean (SD)	1.06 (0.21)

Abbreviation: SD, standard deviation.

**FIGURE 17:**
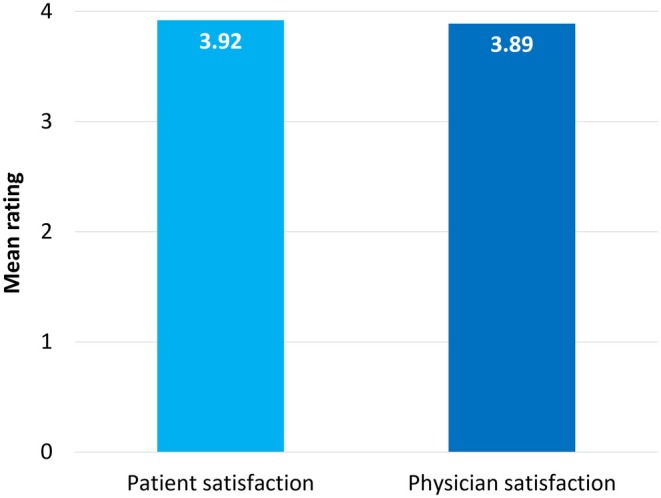
Mean patient and physician satisfaction with the result. Assessments were made 1–3 months posttreatment on a scale of 1 (poor) to 4 (excellent). *N* = 253.

**FIGURE 18:**
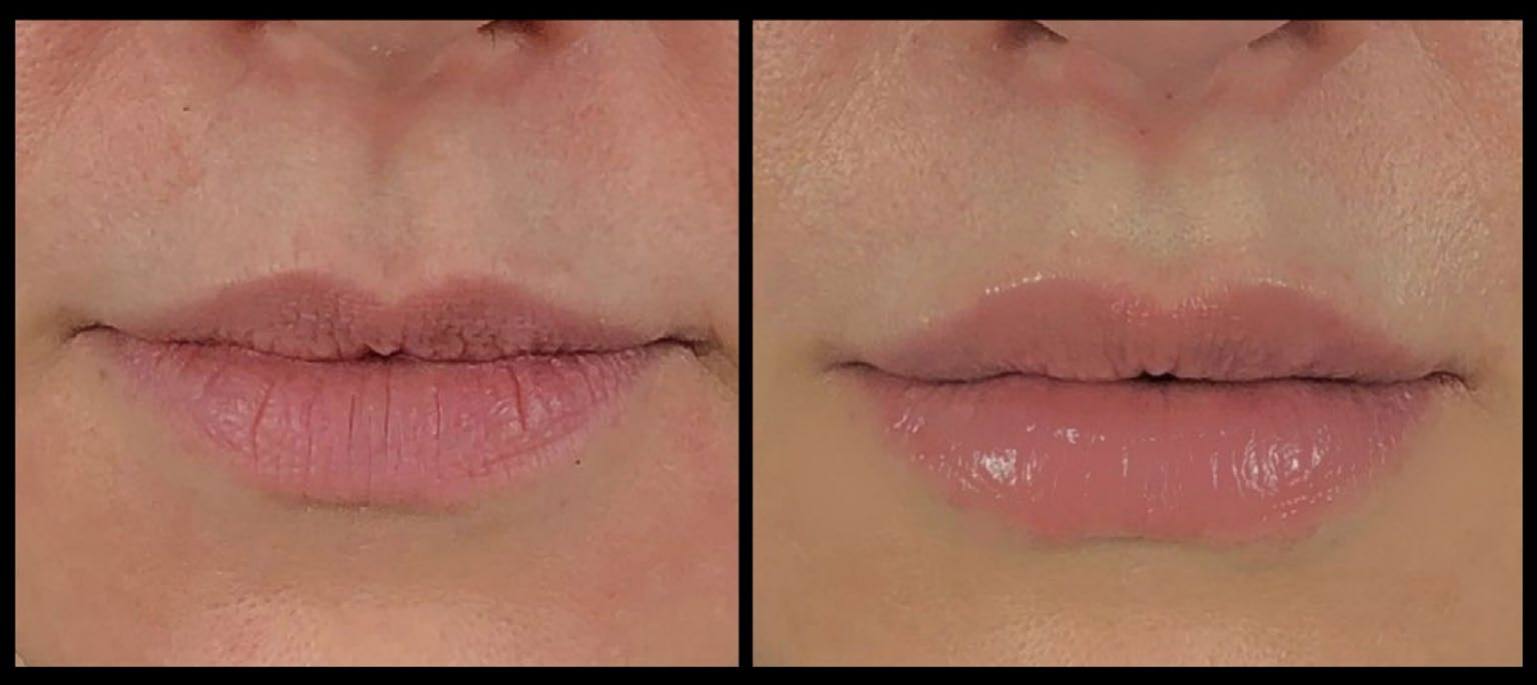
Treatment using the MV Lip technique: Caucasian female. A 49‐year‐old Caucasian female treated using 1 mL of hyaluronic acid filler. She is shown preinjection (left) and 1‐month postinjection (right). MV, Multi Vector.

**FIGURE 19:**
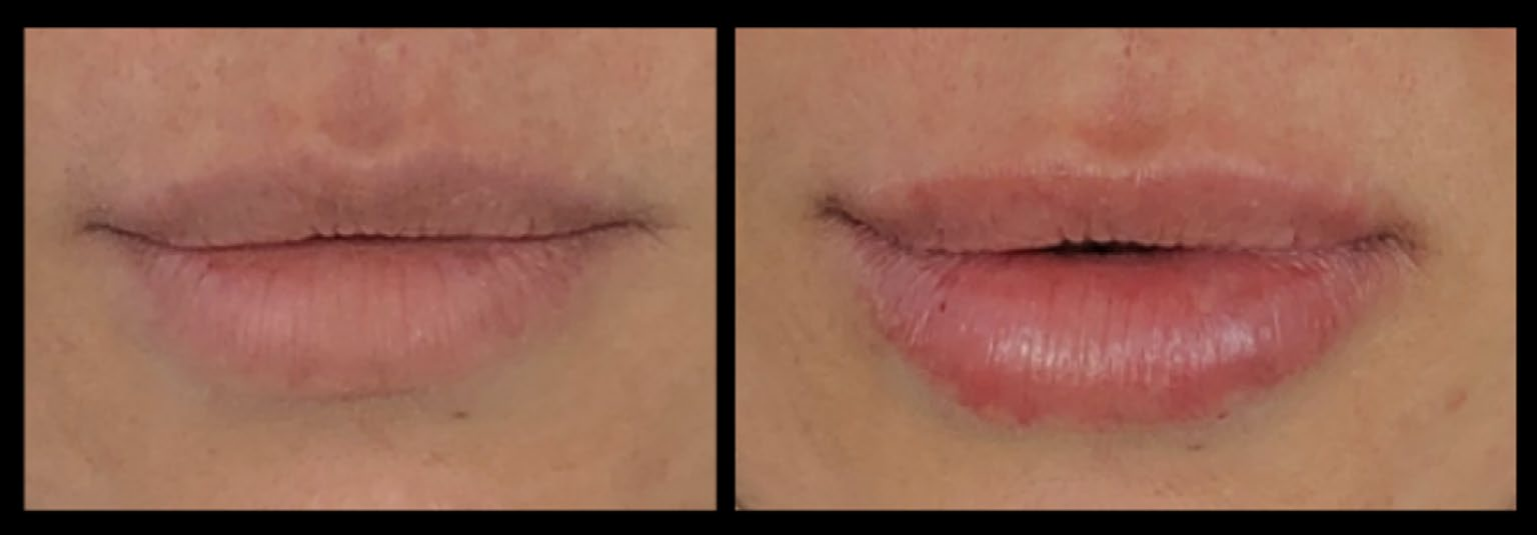
Treatment using the MV Lip technique: Asian female. A 46‐year‐old Asian female treated using 1 mL of hyaluronic acid filler. She is shown preinjection (left) and 1‐month postinjection (right). MV, Multi Vector.

**FIGURE 20:**
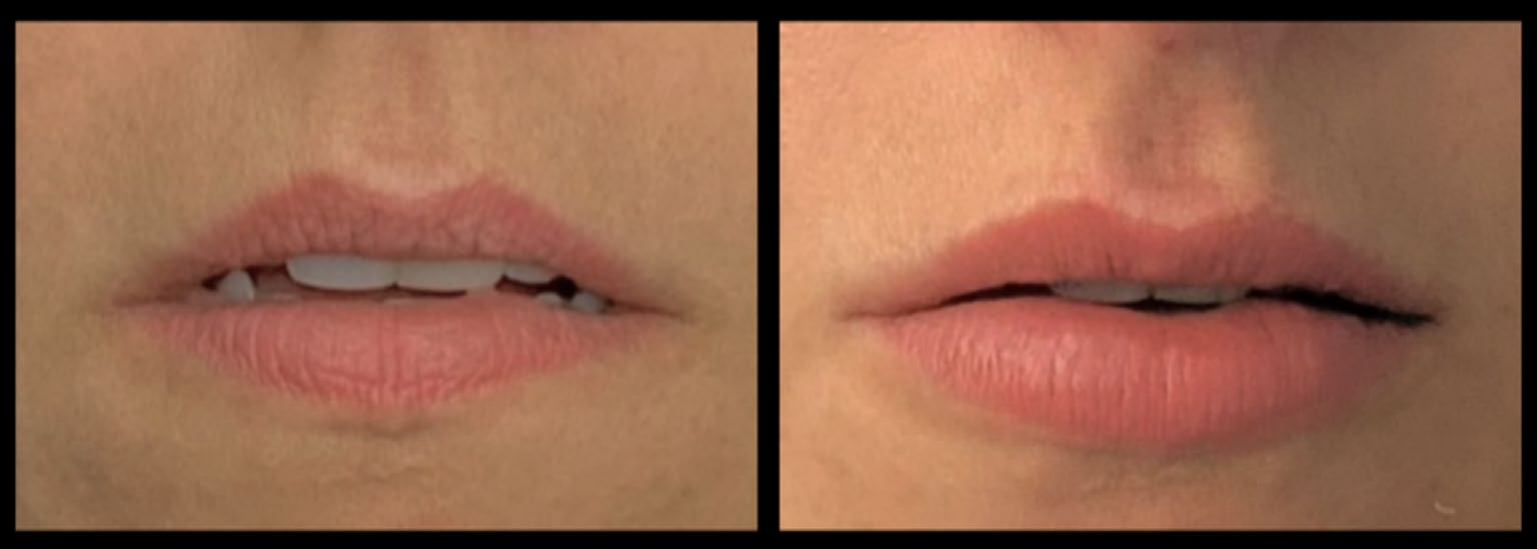
Treatment using the MV Lip technique: Hispanic female. A 45‐year‐old Hispanic female treated using 1 mL of hyaluronic acid filler. She is shown preinjection (left) and 3‐months postinjection (right). MV, Multi Vector.

**FIGURE 21:**
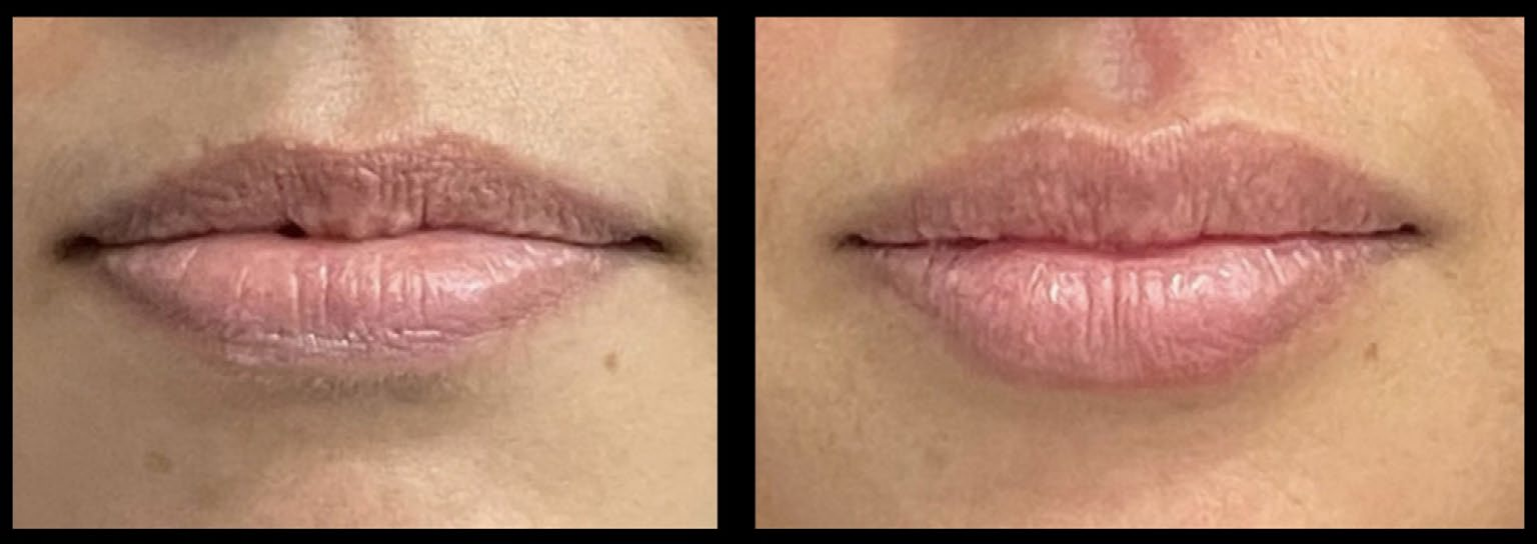
Treatment using the MV Lip technique: Black female. A 39‐year‐old Black female treated using 1 mL of hyaluronic acid filler. She is shown preinjection (left) and 2‐months postinjection (right). MV, Multi Vector.

**FIGURE 22:**
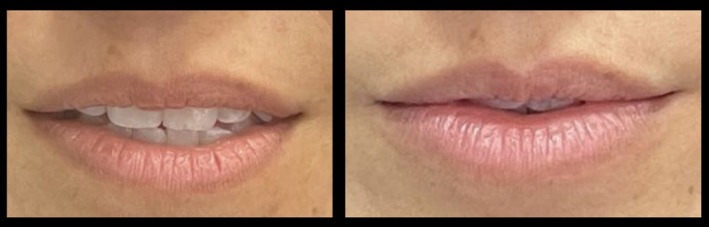
Treatment using the MV Lip technique: Hispanic female. A 45‐year‐old Hispanic female treated using 1 mL of hyaluronic acid filler. She is shown preinjection (left) and 2‐months postinjection (right). This case demonstrates the utility of the method in increasing lip fullness and improving overall balance. MV, Multi Vector.

**FIGURE 23:**
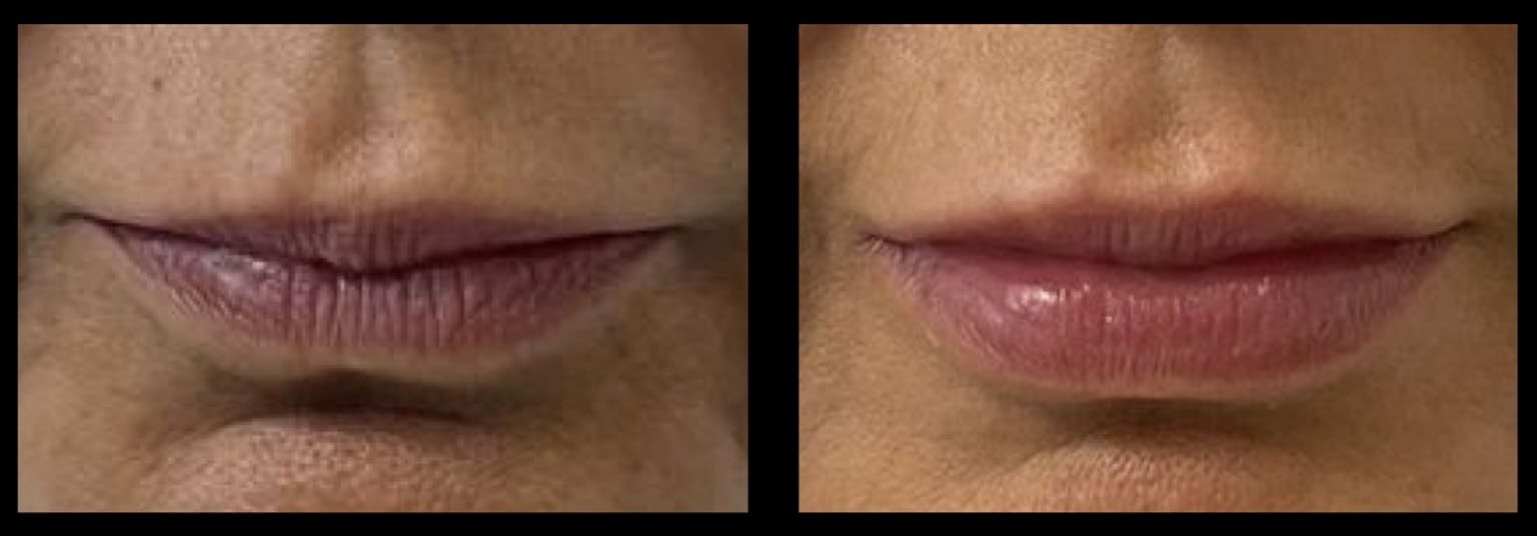
Treatment using the MV Lip technique: Older female. A 65‐year‐old Hispanic female treated using 1 mL of hyaluronic acid filler. She is shown preinjection (left) and 3‐months postinjection (right). The naturalness of the result in this older patient demonstrates the wide clinical utility of the method. MV, Multi Vector.

Included subjects were treated in the lips using a mean of 1.06 ± 0.21 mL of VYC‐17.5L. When patients were asked to rate their satisfaction with the result on a scale of 1 (poor) to 4 (excellent), the mean score was 3.92 ± 0.28 (range: 2–4) (Figure 17). The injecting physicians gave a mean score of 3.89 ± 0.31 (range: 2–4) using the same scale. Example pre‐ and posttreatment photographs are provided in Figures 18, 19, 20, 21, 22, 23.

AEs were minor, with 40 patients (15.8%) experiencing edema and 28 (11.1%) reporting bruising. There were no major complications, including zero recorded instances of vascular occlusion.

## 4 Discussion

We have developed the novel MV Lip technique for aesthetic treatment of the lips using an HA filler, centered on filling geometric shapes. It incorporates injection vectors that are either linear or expansion‐based (bolus). The safety and effectiveness of the method have been demonstrated in 253 female patients treated by five different physicians in centers across Brazil. Levels of patient satisfaction with results were high.

Importantly, the participant group was highly diverse, which is particularly relevant with regard to lip treatment given the variations in normal proportions across different ethnicities [4]. Indeed, aesthetic ideals may vary substantially according to both ethnic background and cultural preferences, and it is therefore notable that the present study confirms safety and effectiveness across the wide range of different ethnicities found in Brazil. Moreover, patient ages varied widely, with the oldest subject being 80 years old. This suggests that our method is sufficiently versatile to be effective with all types of female lips—accommodating variations based on increasing the vertical dimensions, elongating the width, improving contour and projection, accentuating the tubercles, and symmetrizing the lips, according to patient needs. The MV Lip technique is defined by anatomical landmarks and is therefore fully individualizable.

Other approaches to lip rejuvenation and augmentation with HA fillers have been published [14, 15, 16, 17, 18, 19, 20, 21, 22, 23, 24, 25, 26, 27], and all can yield excellent results, but we believe that none has the comprehensiveness and versatility of our method. Moreover, the MV Lip technique is more intuitive, has a straightforward patient assessment process, provides a complete and easily understood treatment approach, and is highly trainable—thus making it particularly well suited for less experienced injectors.

We must also specifically note that there have been previous papers describing vertical linear injections in lip augmentation. For example, the “lip tenting” technique proposed a standardized distribution of vertical injections across the upper and lower lips [26]. More recently, Vitale et al. [27] further developed the vector‐based concept, applying linear retrograde injections from a single dermal entry point. Both approaches advance the understanding of structured and vector‐based injections. However, the MV Lip technique expands this concept by employing multiple individual entry points, with each retrograde injection specifically designed to generate the effect of a distinct vector and its targeted impact on the dermis or vermilion. Furthermore, the MV Lip technique incorporates geometric patterns and strategic deposition in both the dermis of the upper lip and the subcutaneous plane of the lower lip to optimize contour and volume. Taken together, this demonstrates the originality and clinical value of our approach, while also reinforcing the importance of vector‐based injections as a maturing area of aesthetic medicine.

In the current analysis, all treatments were performed using a single HA filler, VYC‐17.5L. This product has the necessary rheological and physicochemical properties for injection into the lips [5]. In particular, it has a medium G′, medium levels of cohesivity to allow for molding, and good tissue integration [5]. VYC‐17.5L also has proven safety and effectiveness in treatment of the lips [8, 9]. Nonetheless, the MV Lip technique can and has been successfully employed with other HA filler products that have the appropriate properties.

All AEs were minor and short‐lived, representing the normal sequelae of this type of treatment (edema and bruising). Anecdotally, patients noted minimal pain. Importantly, there were no major complications throughout 9–12 months of follow up.

Most injections were performed using a needle to facilitate precise targeting of the required anatomical layer. Although the superior and inferior labial arteries show high levels of interpatient variability in their path and depth [28], ultrasound imaging studies have suggested that they are most frequently located in the mucosal/submucosal planes—and only rarely in the subcutaneous plane (≤ 5%) [11, 12]. Our technique primarily targets the latter. We also favor the use of aspiration as an additional safety check every time the needle is inserted. We recorded no instances of vascular occlusion among the 253 patients in the current dataset, suggesting that the method successfully minimized this risk. Nonetheless, we would always advise practitioners to gain sufficient training and exercise particular care when treating the lips with fillers.

The MV Lip technique was developed to align with contemporary beauty standards. Aesthetic ideals are typically characterized by harmonious balance and symmetry, with the lower lip slightly fuller than the upper lip, in a ratio of approximately 1.6:1 among Caucasian females [4]. However, this ideal ratio may vary for individual patients, for example, due to ethnic, cultural, or personal considerations; because the MV Lip technique is based on anatomy, it can be tailored accordingly, and is therefore completely “inclusive.”

In addition, a well‐defined Cupid's bow and a distinct vermilion border contribute to a youthful and natural appearance. The outer limit of the lips should align with the inner edges of the irises. Furthermore, their volume should be evenly distributed, with gentle tapering toward the corners, allowing the lips to complement the overall facial structure while maintaining a natural look that aligns with individual features. In addition, the upper and lower tubercles should interlock, providing movement and curvature, while preserving a natural appearance.

The use of a needle rather than a cannula enables precise sculpting in alignment with these beauty standards. In our hands, this device offers greater control and accuracy, allowing meticulous enhancement of lip shape and volume, and facilitating enhanced definition of Cupid's bow and the vermilion border—crucial elements in achieving symmetry and balance. The MV Lip technique not only respects natural lip anatomy but also allows for customization according to individual patient characteristics, ensuring that enhancements harmonize with overall facial structure.

Importantly, reproducibility is central to the clinical application of the method. The proposed geometric figures serve not only as practical injection guidance but also as diagnostic tools to identify areas with greater or lesser treatment needs. A thorough understanding of the effect of each vector increases the likelihood of consistent and predictable results. Nevertheless, systematic training and structured practice are essential. Four of the co‐authors of the present paper learned the technique directly from the senior author, and are now successfully reproducing it in daily practice as well as teaching the method in workshops across Brazil. Thus, the learning curve is highly manageable with appropriate guidance and hands‐on training.

We should acknowledge the limitations of our study. In particular, it was a retrospective and noncomparative analysis, and it would be interesting to compare the MV Lip technique with other methods in a prospective randomized trial. Nonetheless, the patient group was large and sufficiently diverse in age and ethnicity to suggest that the safety and effectiveness data could be extrapolated to other settings. We also acknowledge that the patients treated in this study were all female. However, the lips are among the lowest priority areas for male patients [29], and the key aesthetic parameters would, of course, be different.

In conclusion, the MV Lip technique offers a versatile, customizable, but also highly reproducible method for the aesthetic improvement of female lips across a wide range of ages and ethnicities. It was safe and effective and led to high levels of patient satisfaction in a group of more than 250 individuals treated with a single HA filler. This technique has the potential to further advance nonsurgical treatment of the perioral area.

## Author Contributions

The study was conceived and designed by Marcel Vinícius de Aguiar Menezes. All authors were involved in data acquisition and analysis, and in drafting the manuscript. All authors gave their final approval of the version to be published and agree to be accountable for all aspects of the work.

## Ethics Statement

The authors confirm that the ethical policies of the journal, as noted on the journal's author guidelines page, have been adhered to. The study was conducted in accordance with the Declaration of Helsinki, and all patients provided written informed consent prior to treatment.

## Consent

All of the patients whose photographs are used in this publication provided written informed consent.

## Conflicts of Interest

Marcel Vinícius de Aguiar Menezes is a speaker for AbbVie. Fernanda Waehneldt Pires Penna is a speaker for AbbVie. All other authors report nothing to disclose.

## Supporting information


**Video S1:** The Multi Vector Lip technique.

**Video S2:** Upper lip: vermilion eversion, increasing lip height, and soft contouring.

**Video S3:** Upper lip: improving fullness and providing rejuvenation with soft volumization.

**Video S4:** Upper lip: contouring laterally.

**Video S5:** Upper lip: defining Cupid's bow.

**Video S6:** Upper lip: defining the philtral column and Glogau–Klein point.

**Video S7:** Upper lip: supporting the cutaneous part and softening the transition (needle).

**Video S8:** Upper lip: supporting the cutaneous part and softening the transition (cannula).

**Video S9:** Lower lip: tubercle definition, vermilion eversion, and increasing vermilion show.

**Video S10:** Lower lip: volumizing the inferior tubercle.

**Video S11:** Lower lip: supporting the medial part.

## Data Availability

The data that support the findings of this study are available from the corresponding author upon reasonable request.
